# Predicting diagnosis of Parkinson's disease: A risk algorithm based on primary care presentations

**DOI:** 10.1002/mds.27616

**Published:** 2019-02-08

**Authors:** Anette Schrag, Zacharias Anastasiou, Gareth Ambler, Alastair Noyce, Kate Walters

**Affiliations:** ^1^ University College London Institute of Neurology University College London London UK; ^2^ University College London Department of Statistical Science University College London London UK; ^3^ University College London Department of Primary Care & Population Health University College London London UK

**Keywords:** algorithm, diagnosis, Parkinson's disease, prodromal, risk, risk calculator

## Abstract

**Background:**

Diagnosis of Parkinson's disease (PD) is typically preceded by nonspecific presentations in primary care.

**Objectives:**

The objective of this study was to develop and validate a prediction model for diagnosis of PD based on presentations in primary care.

**Setting:**

The settings were general practices providing data for The Health Improvement Network UK primary care database.

**Methods:**

Data from 8,166 patients aged older than age 50 years with incident diagnosis of PD and 46,755 controls were analyzed. Likelihood ratios, sensitivity, specificity, and positive and negative predictive values for individual symptoms and combinations of presentations were calculated. An algorithm for risk of diagnosis of PD within 5 years was calculated using multivariate logistic regression analysis. Split sample analysis was used for model validation with a 70% development sample and a 30% validation sample.

**Results:**

Presentations independently and significantly associated with later diagnosis of PD in multivariate analysis were tremor, constipation, depression or anxiety, fatigue, dizziness, urinary dysfunction, balance problems, memory problems and cognitive decline, hypotension, rigidity, and hypersalivation. The discrimination and calibration of the risk algorithm were good with an area under the curve of 0.80 (95% confidence interval 0.78‐0.81). At a threshold of 5%, 37% of those classified as high risk would be diagnosed with PD within 5 years and 99% of those who were not classified as high risk would not be diagnosed with PD.

**Conclusion:**

This risk algorithm applied to routine primary care presentations can identify individuals at increased risk of diagnosis of PD within 5 years to allow for monitoring and earlier diagnosis of PD. © 2019 The Authors. *Movement Disorders* published by Wiley Periodicals, Inc. on behalf of International Parkinson and Movement Disorder Society.

A diagnosis of Parkinson's disease (PD) is made when classical features of slowness combined with other features such as rigidity, tremor, and postural instability are present.^1^ However, clinical symptoms leading to presentation in primary care typically occur several years before a diagnosis is made.^2^, ^3^, ^4^, ^5^, ^6^, ^7^, ^8^ These presentations provide an opportunity to identify those at increased risk of diagnosis of PD, allowing for earlier diagnosis with more effective treatment to improve quality of life^9^ as well as enrollment into clinical trials with potential neuroprotective medications. A number of approaches using individual or combined risk and prediagnostic features to identify higher risk individuals have been proposed, but these are limited by availability of resources (eg, investigations such as transcranial sonography) or rarity of the risk or prediagnostic feature (eg, presence of rapid eye movement sleep behavior disorder or genetic risk factors).^10^, ^11^ In addition, all of these approaches require active screening for risk and prediagnostic features. The Movement Disorders Society Research criteria for prodromal PD combine the published likelihood ratios of readily available risk and presenting factors and of more specific investigations such as dopamine transporter (DAT) imaging.^12^ However, these were derived from multiple individual studies and are designed for active screening of populations; predictive tools to identify those in the prodromal phase of PD from primary care presentations are lacking. We here report the development of a risk model to (1) identify patients with possible prediagnostic PD for participation in future research and (2) aid in the earlier recognition of PD as a cause for symptoms patients present with in primary care.

## Methods

### Study Design and Data Source

We used data from a previous case‐control study identifying prediagnostic features of PD that occurred significantly more often in patients with a later diagnosis of PD than in matched controls.^3^ In brief, data were derived from a large primary care database in the United Kingdom, The Health Improvement Network, which holds pseudanonymized longitudinal medical records for more than 11 million individuals registered with more than 500 general practices in the United Kingdom. Information on symptoms, diagnoses, interventions, and referrals to secondary care are electronically recorded as read codes, a hierarchical coding system used in U.K. primary care, which map on to International Classification of Diseases, 10th Revision codes.^13^ The Health Improvement Network data are representative of the U.K. general practice population in terms of demographics and frequency and type of consultations requested by patients, and electronically coded diagnoses have been shown to be accurate.^14^ The Health Improvement Network data collection scheme was approved by UK South East National Health Service multicenter research ethics committee, and the scientific review committee approved the present study.

### Study Population

We identified all individuals who had a read code diagnosis of PD and at least 2 antiparkinsonian drug prescriptions. A similar method for the identification of people with PD has been validated in another large primary care database in the United Kingdom.^15^ Diagnostic read codes for PD were identified using published methods (Appendix).^16^ The earliest date of Read code diagnosis or antiparkinsonian drug prescription was taken as the index date. Individuals with a diagnosis of PD before the age of 50 years were excluded, as were those with secondary parkinsonism, dementia before PD diagnosis, drug‐induced parkinsonism, or schizophrenia (because these individuals are likely to have had substantial exposure to dopamine antagonist drugs). Six times as many controls as cases with similar distribution of age, gender, and registration period at the date of a general practice consultation (index date) were randomly selected (frequency matching) using a random sampling routine. Individuals were included only if they had at least 1 year of data before the index date. This inclusion criterion ensured that individuals had at least 1 year between registration with the GP practice and diagnosis of PD, which limits the possibility of inclusion of patients with PD diagnosed previously but first recorded by the new GP during the patient registration period.^17^

### Data Extraction

Symptoms initially included in the analysis were first presentations of late‐onset (>50 years of age) anxiety and depression, fatigue, apathy, insomnia, balance impairment, dizziness, hypotension, anosmia, hypersalivation, constipation, urinary dysfunction, erectile dysfunction, memory problems, neck pain or stiffness, shoulder pain or stiffness, rigidity, tremor, and cognitive decline. All symptoms were defined using read code lists.^16^ In addition, we used prescriptions for anxiolytics, antidepressants, drugs for constipation, and hypnotics and drugs for erectile dysfunction to identify symptoms of anxiety, depression, constipation, insomnia, and erectile dysfunction, respectively. For the variables anxiety and depression with onset >50 years of age, those with a record of anxiety or depression before age 50 years were treated as missing. As symptoms coded shortly after registration with the GP potentially represent prevalent and not new health issues, the exclusion period was 1 year after GP registration for anxiety or depression and 6 months for all other symptoms.

### Analysis

We restricted analysis to the first presentations within 5 years of diagnosis of PD or index date in controls. We calculated the percentage of patients with each presentation in patients and controls. To allow for comparison with previously published data on risk associated with the examined prodromal features,^12^ we also calculated sensitivity, specificity, positive and negative likelihood ratios, positive predictive values (PPVs) and negative predictive values for each presentation, and smoking (current, exsmoker, or never) and alcohol consumption (current, former, or never). PPVs were calculated using Bayes’ theorem,^18^ where posterior odds of disease = prior odds × likelihood ratio. For prior odds, we used a prevalence rate of PD for older than the age of 50 years of 1,400 per 100,000.^19^ In addition, we calculated PPV for all 2‐symptom combinations of 2 individually significant symptom presentations.

Univariate logistic regression was used to examine the differences between cases and controls in each prediagnostic presentation as well as in smoking status and alcohol consumption (as they are known to have negative association with risk of PD), adjusted for age group, gender, and index date.

### Development of the Risk Model

We then separated the dataset into a development and a validation sample using random sampling of individuals, comprising 70% and 30% of the original dataset, respectively. Using the development sample, symptoms independently associated with PD diagnosis with a *P* value < .1 were entered into a backward multivariate logistic regression analysis with PD diagnosis as the dependent variable with a *P* removal = .05. We combined the variables anxiety and depression with onset >50 years (present if either for the first time occurred after the age of 50 years) because these symptoms are often comorbid^12^ and therefore not independent. We then applied the final model to the validation sample and compared the performance in the development and validation samples. As this study is a case‐control study, the estimated intercept is too high and leads to an overestimate of the actual risk of PD in the population. Therefore, corrected intercepts were calculated for each age group/gender combination (see Box 1) so that the average risk predicted by the model reflects the age‐ and gender‐specific prevalence of PD^20^ (Table S3). The discriminatory ability of the final model was quantified through an area under a receiver operating characteristic curve with sensitivity and specificity.^21^ Calibration was assessed by graphically comparing the observed and predicted values within decile groups of predicted risk. (The same values used for the Hosmer‐Lemeshow test; the *P* value is not reported because this test tends to produce significant results in large samples, even when the observed and predicted values are very close.^22^) The model's calibration was also assessed using the calibration slope.^23^ All analyses were performed using Stata (version 14; StataCorp, College Station, Texas).

BOX 1. 1 The risk model constructed to calculate predicted risk of PD is the following:

$${\text{Patient}}^{’}s\;\text{risk of}\;\mathrm{PD}=\frac{1}{1+\exp\left(-\text{patien}t^{\prime }s\;\text{risk score}\right)};$$

where Patient^′^s risk score = *b*_1_×I(Tremor)+ *b*_2_×I(Constipation) + *b*_3_×I(Depression and/or Anxiety) + *b*_4_×I(Fatigue) + *b*_5_×I(Dizziness) + *b*_6_×I(Urinary Dysfunction) + *b*_7_×I(Balance Problems) + *b*_8_×I(Memory Problems) + *b*_9_×I(Hypotension) + *b*_10_×I(Rigidity) + *b*_11_×I(Cognitive Decline) + *b*_12_×I(Hypersalivation) + *b*_13_×I(Exsmoker) + *b*_14_×I(Current Smoker) + *b*_15_×I(age = 50 and male) +*b*_16_×I(age = 60 and male) + *b*_17_×I(age = 70 and male) + *b*_18_×I(age = 80 and male) + *b*_19_×I(age = 90 and male) + *b*_20_×I(age = 50 and female)+ *b*_21_×I(age = 60 and female) + *b*_22_×I(age = 70 and female) + *b*_23_×I(age = 80 and female) + *b*_24_×I(age = 90 and female)

Using an example for the final model, a 70‐year‐old male who has constipation, tremor, depression, anxiety, and urinary dysfunction has a predicted risk of Parkinson's disease of 67% (61%‐72%). Based on Tables 2 and Supporting Information Table S3, the patient's risk score = 0.50 + 4.58 + 0.43 + 0.52‐5.32 = 0.71.

The patient's risk of PD = 1/(1 + exp(−0.71)) = 0.67 or 67%.

**Table 1:** Characteristics of patients with Parkinson's disease and controls

Characteristic	Parkinson's disease, n = 8,166	Controls, n = 46,755
Gender, n (%)		
Men	4,859 (59.5)	27,684 (59.21)
Women	3,307 (40.5)	19,071 (40.79)
Age at index date, 10‐year age bands, n (%)		
50‐60	649 (7.95)	3,740 (8.00)
60‐70	1,855 (22.72)	10,986 (23.50)
70‐80	3,437 (42.09)	20,324 (43.47)
80‐90	2,039 (24.97)	11,113 (23.77)
90+	186 (2.28)	892 (1.72)
Years of follow‐up, mean (SD)	4.1 (1.27)	4.0 (1.33)
Smoking status, n (%)		
Never	4,396 (53.83)	20,589 (44.04)
Past	2,076 (25.42)	12,196 (26.08)
Present	711 (8.71)	7,594 (16.24)
Data missing	983 (12.04)	6,376 (13.64)
Alcohol consumption, n (%)		
Never	1,042 (12.76)	5,751 (12.30)
Past	371 (4.54)	1,743 (3.73)
Present	5,240 (64.17)	29,217 (62.49)
Data missing	1,513 (18.53)	10,044 (21.48)

## Results

**Figure 1:**
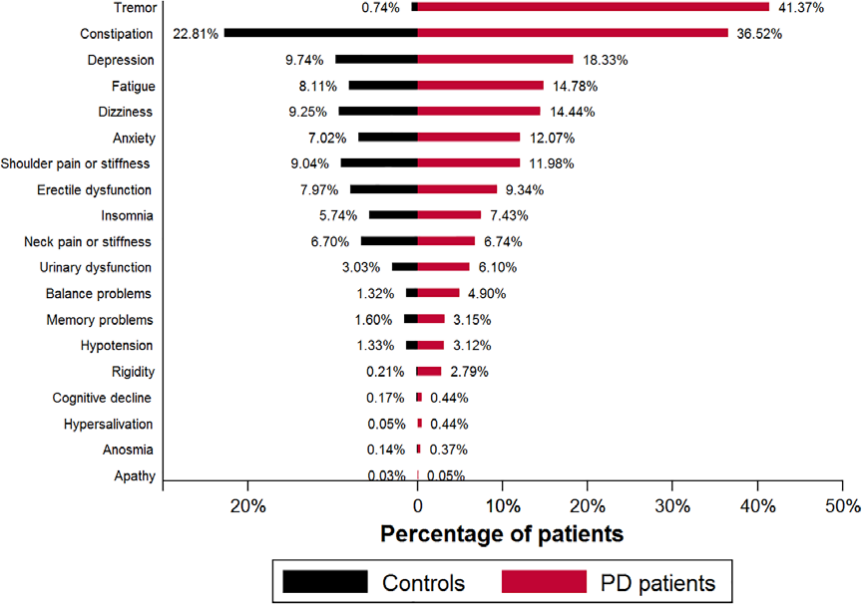
Percentage of individual symptom presentations in controls and PD patients. [Color figure can be viewed at wileyonlinelibrary.com]

A total of 8,166 individuals with PD and 46,755 controls were included in the study (Table 1).^3^ Figure 1 shows the frequency of the potential prediagnostic features in the PD group when compared with the control group within 5 years before diagnosis. The most common prediagnostic symptom of PD within 5 years before diagnosis was tremor, with 41% of individuals reporting symptoms to their GP compared with less than 1% of controls. Constipation occurred in 37% versus 23% in controls, depression in 18% versus 10%, fatigue in 15% versus 8%, dizziness in 14% versus 9%, anxiety in 12% versus 7%, and shoulder stiffness or pain in 12% versus 9%. In univariate logistic regression, all presentations except apathy and neck pain/stiffness had a significant association with PD diagnosis at a significance level of *P* < .05 (Supporting Information Table S1). Sensitivity, specificity, positive and negative likelihood ratios, and positive and negative predictive values of each individual presentation are given in Supporting Information Table S2.

**Table 2:** Multivariable logistic regression analysis for association of symptom presentations and risk factors with diagnosis of Parkinson's disease, adjusted for age group and gender in the development sample

Presentation/Risk factor	Regression coefficient	*P* value	95% CI	OR	95% CI
Intercept	−2.47	<.001	−2.54 to −2.41		
Tremor	4.58	<.001	4.41 to 4.74	97.21	82.55 to 114.49
Constipation	0.50	<.001	0.41 to 0.58	1.64	1.51 to 1.79
Depression and/or anxiety	0.43	<.001	0.32 to 0.53	1.53	1.38 to 1.69
Fatigue	0.38	<.001	0.25 to 0.51	1.46	1.28 to 1.67
Dizziness	0.15	.025	0.02 to 0.29	1.17	1.02 to 1.33
Urinary dysfunction	0.52	<.001	0.34 to 0.70	1.68	1.40 to 2.00
Balance problems	1.39	<.001	1.17 to 1.62	4.03	3.22 to 5.04
Memory problems	0.34	.018	0.06 to 0.63	1.41	1.06 to 1.87
Hypotension	0.46	.001	0.18 to 0.74	1.58	1.19 to 2.10
Rigidity	2.49	<.001	2.11 to 2.88	12.10	8.22 to 17.83
Cognitive decline	1.00	.001	0.39 to 1.61	2.72	1.48 to 5.01
Hypersalivation	1.99	<.001	1.08 to 2.91	7.35	2.95 to 18.28
Smoking status					
Never	0	<.001		1	
Past	−0.33		−0.42 to −0.24	0.72	0.66 to 0.78
Present	−0.86		−0.98 to −0.73	0.42	0.37 to 0.48

95% CI, 95% confidence interval.

### Development of the Model

**Figure 2:**
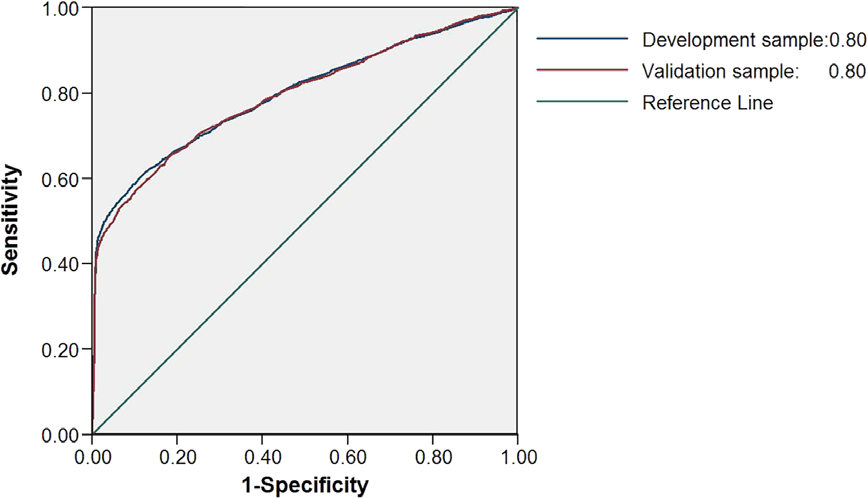
Receiver operating characteristic curves for the prediction of PD for the model with all statistically significant prediagnostic symptom presentations of PD, smoking status, gender, and age group in the development and validation datasets. [Color figure can be viewed at wileyonlinelibrary.com]

In the development sample following multivariate analysis, the following presentations remained significant predictors of diagnosis of PD: tremor, hypersalivation, rigidity, memory problems, urinary dysfunction, fatigue, hypotension, dizziness, constipation, cognitive decline, balance problems, depression and/or anxiety, and smoking status (Table 2). Insomnia, anosmia, and shoulder pain were no longer significantly associated with later diagnosis of PD in the multivariate analysis. For the model construction, Supporting Information Table S3 shows the factors to be subtracted in the risk model to adjust for patient age and gender. In the final model the area under the curve (AUC) was 0.80 (95% confidence interval [CI] 0.78‐0.81; Fig. 2). Validation of the model in the validation sample showed a similar AUC of 0.80 (95%CI 0.78‐0.81; *P* = .69). The calibration slope was close to 1 in the validation sample, suggesting good calibration (Supporting Information Figure S1).

As tremor had a very high predictive value, we also repeated the analysis without the inclusion of tremor (Supporting information Tables 4 and 5 and Figures 2 and 3). This showed similar results for the other variables, albeit with a lower AUC of 0.66 (95%CI 0.65‐0.67).

**Table 3:** Risk classification using the Parkinson's disease risk algorithm for all factors in the final model

Cut off for high risk, %	Sensitivity (95% CI)	Specificity (95% CI)	PPV (95%CI)	NPV (95%CI)
1	55.86 (54.63‐57.07)	91.98 (91.70‐92.26)	9.00 (8.67‐9.35)	99.32 (99.30‐99.34)
5	43.48 (42.26‐44.70)	98.94 (98.83‐99.05)	36.88 (34.50‐39.33)	99.20 (99.18‐99.21)
10	40.84 (39.63‐42.05)	99.18 (99.08‐99.27)	41.48 (38.66‐44.36)	99.16 (99.14‐99.18)
20	32.41 (31.27‐33.57)	99.36 (99.27‐99.44)	41.81 (38.61‐45.08)	99.04 (99.03‐99.05)

95% CI, 95% confidence interval; NPV, negative predictive values; PPV, positive predictive values.

### Risk Classification

Using a range of possible cut‐offs to indicate high risk for PD, the specificity of the risk algorithm was high and there was a high negative predictive value, but lower sensitivity and PPV (Tables 3 and 4). For example, if we chose a threshold of 5% to split the patients into high‐risk and low‐risk groups based on their predicted risk, the specificity was 98.94%, negative predictive value was 99.20%, sensitivity was 43.48%, and PPV was 36.88%.

**Table 4:** Risk classification using the Parkinson's disease risk algorithm for all factors in the final model (without tremor)

Cut off for high risk, %	Sensitivity (95% CI)	Specificity (95% CI)	PPV (95%CI)	NPV (95%CI)
1	47.51 (46.28‐48.74)	71.03 (70.55‐71.49)	2.28 (2.21‐2.34)	98.96 (98.94‐98.99)
5	4.56 (4.07‐5.10)	99.35 (99.26‐99.43)	9.08 (7.77‐10.58)	98.65 (98.647‐98.66)
10	1.99 (1.66‐2.36)	99.83 (99.78‐99.87)	13.90 (10.68‐17.90)	98.625 (98.62‐98.63)

95% CI, 95% confidence interval; NPV, negative predictive values; PPV, positive predictive values.

## Discussion

We used routinely collected primary care data to develop a risk model for diagnosis of PD within 5 years following the first presentation with possible prediagnostic features. It provides a clinical tool for use in primary care, which does not require additional testing but allows one to identify individuals older than 50 years for monitoring or early referral for suspected diagnosis of PD. It therefore has the potential to allow for earlier diagnosis of PD, which is typically delayed by >1 year,^24^ leading to delayed treatment and reduced quality of life^9^ and to refer and initiate treatment early and effectively. In addition, it provides the opportunity to identify individuals in the general population for studies of the prodromal phase of PD, which so far have required the identification of rare factors associated with high risk (eg, gene carriers of leucine‐rich repeat kinase 2 [*LRRK2*] mutations), expensive investigations, or active screening of large volunteer cohorts. As this tool does not require any additional investigations other than information already collected in routine health care records and as it is based in primary care, it provides researchers with a method to identify individuals with increased risk on a large scale and from a representative general population. Although further examination, monitoring, or testing is likely to be necessary for inclusion in further biomarker or treatment trials (eg, neurological examination, imaging, or genetic testing), this approach overcomes some of the challenges in this field, including the ethical dilemma of identifying individuals at risk who do not have troublesome symptoms requiring treatment, as individuals are considered at a time when they are seeking medical help for their symptoms.

Our results for individual prediagnostic features are comparable to those that were also included in the recently published Movement Disorders Society research criteria for prodromal PD,^12^ although the predictive factors we included differed because of the nature of the studies (eg, the Movement Disorders Society criteria included investigational results and the present data include a wider range of clinical features). These criteria are the current standard for classification of risk of PD as they were based on an evidence based systematic review of risk reported in the literature. However, the classification is necessarily limited by the pooling of different studies and does not account for possible cooccurrence of symptoms, for example, constipation and urinary symptoms. The current study used a single large population to derive likelihood ratios, which were adjusted for each other in multiple regression analysis, thus reducing this limitation. It also uses solely clinical features that are easily attainable in routine clinical settings. Nalls and colleagues^11^ also used a multivariate regression analysis approach to develop a risk model for diagnosis of PD in already established cases and included a genetic risk score. Apart from the genetic score, the University of Pennsylvania Smell Identification Test (UPSIT) together with age, gender, and family history, independently contributed to prediction of PD in established disease, with an AUC of 0.60 (95% CI 0.56‐0.65) for demographic features, 0.64 (95% CI 0.59‐0.69) for the genetic risk score, 0.90 (95%CI 0.87‐0.93) for the UPSIT score, and 0.92 (95% CI 0.90‐0.95) for the integrated score. In our study of prediagnostic PD, using primary care presentations (without UPSIT scores or genetic testing and not including family history), we were still able to achieve an AUC of 0.80 (95% CI 0.78‐0.81), suggesting that the clinical features are a very effective tool to screen for risk of future PD and that further refinement through additional tests, such as UPSIT or genetic testing, may achieve even higher predictive accuracy. Several studies are currently underway to identify further biomarkers and tools to identify risk of PD for clinical trials, including the Parkinson associated risk syndrome (PARS) study, which has already established that screening with UPSIT is an effective way to stratify for those with makers of PD and those without.^25^, ^26^ The Tübinger Evaluation of Risk Factors for Early Detection of Neurodegeneration (TREND) study established transcranial sonography as a useful screening tool,^27^ and the PREDICT‐PD study has pioneered an internet‐based assessment tool.^28^ Incorporating the current risk model as a first step into these studies is likely to improve their ability to screen from populations in primary care and the general population and select individuals for inclusion into trials. The timeframe of 5 years is longer than the duration of most traditional treatment trials but allows for further enrichment using targeted testing.

It is noteworthy that tremor was the clinical feature with the highest predictive value. This may suggest that some patients may already have diagnosable PD, but even in specialist settings making a diagnosis of PD based on presence of tremor alone (even if typical of PD), is currently not possible according to diagnostic criteria. Although the study was not designed to distinguish those with tremor as a result of early PD from those with tremor as a result of other causes, the tool allows for the incorporation of tremor in the risk prediction algorithm. The algorithm, however, does not require the presence of tremor and even when excluding tremor the algorithm based on the combination of other risk and diagnostic features provided acceptable predictive accuracy (AUC 0.66).

### Study Limitations

This study is likely to have underestimated the incidence of prediagnostic features of PD in patients and controls, as only presentations recorded in health care records by primary care physicians were included rather than all symptoms that may have been present on active screening. In addition, these symptom codes are not strict diagnostic codes but reflect presenting complaints or findings. However, these data are likely to be clinically relevant as only symptoms present when patients sought medical help were included, and the risk of recall bias is low as the information was collected from prospectively collected primary care data. This dataset therefore provides prospective information for clinically important features in primary care, making it a more clinically relevant dataset. Despite this potential underestimation of its true predictive power of the risk score we found that the risk score had good predictive power with an AUC of 0.80, making this a useful tool to identify for those who may benefit from further assessment, and the predictive power is likely to be even higher in prospective active screening programs. This tool may help clinicians to identify those whom they should review carefully for features of PD.

## Author Roles

1) Research project: A. Conception, B. Organization, C. Execution; 2) Statistical Analysis: A. Design, B. Execution, C. Review and Critique; 3) Manuscript: A. Writing of the first draft, B. Review and Critique.

A.S.: 1A, 1B, 1C, 2A, 2B, 3A

Z.A.: 2C, 3B

G.A.: 2A, 2B, 2C, 3B

A.N.: 3B

K.W.: 1B, 3B

## Full financial disclosures for the past 12 months

A.S. has received grant funding from Parkinson's UK, the Economic and Social Research Council, the Department of Health's National Institute for Health Research Biomedical Research Centres, GE Healthcare, the European Commission, and University College London. She has received royalties from Oxford University Press and personal fees from Medtronic. A.N. has received received grant funding from Parkinson's UK, Barts Charity and the Virginia Keiley Benefaction, research support from GKC and honoraria from Profile Pharmaceuticals. K.W. has had grant funding from the National Institute of Health Research and National School of Primary Care Research. Z.A. and G.A. report no disclosures.

## Supporting information


**Table S1** Univariate logistic regression models for association of individual symptom presentations and risk factors with diagnosis of Parkinson's disease adjusted for age‐group, gender and index date.
**Table S2.** LRs, PPV and NPV of individual prediagnostic symptom presentations and risk factors
**Table S3.** Corrected intercepts
**Table *S*4.** Multivariate logistic regression models for association of individual symptom presentations and risk factors (excluding tremor) with diagnosis of Parkinson's disease.
**Table S5.** Factors to be added in the calculation (without tremor) of predicted risk of PD to adjust for age group and gender.
**Table S6.** LRs, PPV and NPV of individual prediagnostic symptom presentations and risk factors separately for the Development and the Validation samples
**Figure S1.** Calibration slopes in the development and validation datasets.
