# Supplementary material for: Predicting diagnosis of Parkinson's disease: A risk algorithm based on primary care presentations
Source: Mov Disord. 2019 Feb 8;34(4):480–6. doi: 10.1002/mds.27616 (PMC6518931; doi:10.1002/mds.27616)
Supplement: Supplementary file 1 — Table S1 Univariate logistic regression models for association of individual symptom presentations and risk factors with diagnosis of Parkinson's disease adjusted for age‐group, gender and index date. Table S2. LRs, PPV and NPV of individual prediagnostic symptom presentations and risk factors Table S3. Corrected intercepts Table S4. Multivariate logistic regression models for association of individual symptom presentations and risk factors (excluding tremor) with diagnosis of Parkinson's disease. Table S5. Factors to be added in the calculation (without tremor) of predicted risk of PD to adjust for age group and gender. Table S6. LRs, PPV and NPV of individual prediagnostic symptom presentations and risk factors separately for the Development and the Validation samples Figure S1. Calibration slopes in the development and validation datasets. [file MDS-34-480-s001.docx]

**Supplementary material**

**Table 1. Univariate logistic regression models for association of individual symptom presentations and risk factors with diagnosis of Parkinson's disease adjusted for age-group, gender and index date.**

|  | **OR** | **p-value** |  | **95% CI** | |
| --- | --- | --- | --- | --- | --- |
| Tremor | 100·66 | <0·001 |  | 88·66 | 114·28 |
| Constipation | 1·98 | <0·001 |  | 1·88 | 2·08 |
| Depression and/or anxiety | 1·94 | <0·001 |  | 1·83 | 2·06 |
| Depression | 2·08 | <0·001 |  | 1·94 | 2·23 |
| Fatigue | 1·97 | <0·001 |  | 1·85 | 2·11 |
| Dizziness | 1·65 | <0·001 |  | 1·54 | 1·77 |
| Anxiety | 1·83 | <0·001 |  | 1·69 | 1·99 |
| Shoulder pain or stiffness | 1·37 | <0·001 |  | 1·28 | 1·48 |
| Erectile dysfunction* | 1·23 | 0·0005 |  | 1·09 | 1·38 |
| Insomnia | 1·32 | <0·001 |  | 1·2 | 1·44 |
| Neck pain or stiffness | 1·01 | 0·879 |  | 0·92 | 1·10 |
| Urinary dysfunction | 2·05 | <0·001 |  | 1·84 | 2·30 |
| Balance problems | 3·77 | <0·001 |  | 3·28 | 4·34 |
| Memory problems | 1·97 | <0·001 |  | 1·7 | 2·29 |
| Hypotension | 2·34 | <0·001 |  | 2·02 | 2·72 |
| Rigidity | 13·97 | <0·001 |  | 10·99 | 17·74 |
| Cognitive decline | 2·53 | <0·001 |  | 1·76 | 3·66 |
| Hypersalivation | 8·71 | <0·001 |  | 5·37 | 14·12 |
| Anosmia | 2·64 | <0·001 |  | 1·74 | 4·02 |
| Apathy | 1·78 | 0·383 |  | 0·49 | 6·54 |
| Smoking status |  |  |  |  |  |
| Never | 1 | <0·001 |  |  |  |
| Past | 0·78 |  |  | 0·74 | 0·83 |
| Present | 0·44 |  |  | 0·40 | 0·47 |
| Alcohol consumption |  |  |  |  |  |
| Never | 1 | 0·0206 |  |  |  |
| Past | 1·17 |  |  | 1·03 | 1·33 |
| Present | 0·99 |  |  | 0·92 | 1·07 |
| *data for men only used |  |  |  |  |  |

**Table 2. LRs, PPV and NPV of individual prediagnostic symptom presentations and risk factors**

|  | **Number of patients with** | | **LR+** | **LR to** | **PPV in** | **NPV in** |
| --- | --- | --- | --- | --- | --- | --- |
|  | **Presentation** | | **(95%CI)** | **(95%CI)** | **percent** | **percent** |
|  | **PD** | **Controls** |  |  | **(95%CI)** | **(95%CI)** |
|  |  |  |  |  |  |  |
| **Total** | 8166 | 46755 |  |  |  |  |
| **Tremor** | 3378 | 344 | 56·22 | 0·591 | 44·39 | 99·17 |
|  |  |  | (50·45 to 62·66) | (0·58 to 0·602) | (41·73 to 47·08) | (99·17 to 99·18) |
| **Constipation** | 2982 | 10664 | 1·6 | 0·822 | 2·22 | 98·85 |
|  |  |  | (1·55 to 1·65) | (0·808 to 0·837) | (2·15 to 2·3) | (98·85 to 98·87) |
| **Depression *** | 1378 | 4205 | 1·88 | 0·905 | 2·6 | 98·73 |
|  |  |  | (1·78 to 1·99) | (0·895 to 0·915) | (2·47 to 2·75) | (98·73 to 98·75) |
| **Fatigue** | 1207 | 3791 | 1·82 | 0·927 | 2·52 | 98·7 |
|  |  |  | (1·72 to 1·94) | (0·919 to 0·936) | (2·38 to 2·68) | (98·7 to 98·71) |
| **Dizziness** | 1179 | 4327 | 1·56 | 0·943 | 2·17 | 98·68 |
|  |  |  | (1·47 to 1·66) | (0·934 to 0·952) | (2·04 to 2·3) | (98·68 to 98·69) |
| **Anxiety **** | 918 | 3076 | 1·72 | 0·946 | 2·38 | 98·67 |
|  |  |  | (1·6 to 1·84) | (0·938 to 0·954) | (2·23 to 2·55) | (98·67 to 98·69) |
| **Shoulder pain/stiffness** | 978 | 4228 | 1·32 | 0·968 | 1·85 | 98·64 |
|  |  |  | (1·24 to 1·41) | (0·96 to 0·976) | (1·73 to 1·97) | (98·64 to 98·66) |
| **Erectile dysfunction ***** | 454 | 2206 | 1·17 | 0·985 | 1·64 | 98·62 |
|  |  |  | (1·06 to 1·29) | (0·976 to 0·995) | (1·49 to 1·8) | (98·62 to 98·63) |
| **Insomnia** | 607 | 2682 | 1·3 | 0·982 | 1·81 | 98·62 |
|  |  |  | (1·19 to 1·41) | (0·976 to 0·988) | (1·66 to 1·96) | (98·62 to 98·63) |
| **Neck pain/stiffness** | 550 | 3134 | 1  (0·92 to 1·1) | 1 | 1·41 | 98·6 |
|  |  |  |  | (0·993 to 1·006) | (1·29 to 1·53) | (98·6 to 98·61) |
| **Urinary dysfunction** | 498 | 1416 | 2·01 | 0·968 | 2·78 | 98·64 |
|  |  |  | (1·82 to 2·22) | (0·963 to 0·974) | (2·52 to 3·06) | (98·64 to 98·65) |
| **Balance problems** | 400 | 619 | 3·7 | 0·964 | 4·99 | 98·65 |
|  |  |  | (3·27 to 4·19) | (0·959 to 0·969) | (4·44 to 5·61) | (98·65 to 98·66) |
| **Memory problems** | 257 | 750 | 1·96 | 0·984 | 2·71 | 98·62 |
|  |  |  | (1·71 to 2·26) | (0·98 to 0·988) | (2·37 to 3·1) | (98·62 to 98·63) |
| **Hypotension** | 255 | 622 | 2·35 | 0·982 | 3·23 | 98·63 |
|  |  |  | (2·03 to 2·71) | (0·978 to 0·986) | (2·81 to 3·71) | (98·63 to 98·63) |
| **Rigidity** | 228 | 96 | 13·6 | 0·974 | 16·18 | 98·64 |
|  |  |  | (10·73 to 17·24) | (0·97 to 0·978) | (13·22 to 19·67) | (98·64 to 98·64) |
| **Cognitive decline** | 79 | 36 | 2·61 | 0·997 | 3·57 | 98·6 |
|  |  |  | (1·76 to 3·87) | (0·996 to 0·999) | (2·44 to 5·2) | (98·6 to 98·61) |
| **Hypersalivation** | 36 | 23 | 8·96 | 0·996 | 11·29  (7·02 to 17·67) | 98·61 |
|  |  |  | (5·31 to 15·11) | (0·995 to 0·998) |  | (98·61 to 98·61) |
| **Anosmia** | 30 | 66 | 2·6 | 0·998 | 3·56 | 98·6 |
|  |  |  | (1·69 to 4) | (0·996 to 0·999) | (2·35 to 5·38) | (98·6 to 98·61) |
| **Apathy** | 4 | 13 | 1·76 | 1 | 2·44 | 98·6 |
|  |  |  | (0·57 to 5·4) | (0·999 to 1) | (0·81 to 7·12) | (98·6 to 98·6) |
| **Smoking status****** | |  |  |  |  |  |
| **Never** | 4398 | 20589 | 1·2 | 0·79 | 1·68 | 98·89 |
|  |  |  | (1·18 to 1·23) | (0·77 to 0·82) | (1·64 to 1·71) | (98·85 to 98·92) |
| **Past** | 2076 | 12196 | 0·956 | 1·02 | 1·34 | 98·57 |
|  |  |  | (0·92 to 0·99) | (1·002 to 1·035) | (1·29 to 1·39) | (98·55 to 98·6) |
| **Present** | 711 | 7594 | 0·56 | 1·11 | 0·74 | 98·45 |
|  |  |  | (0·49 to 0·75) | (1·1 to 1·12) | (0·69 to 0·80) | (98·43 to 98·46) |
| **Alcohol consumption******* | | |  |  |  |  |
| **Never** | 1042 | 5751 | 1 | 1 | 1·4 | 98·6 |
|  |  |  | (0·94 to 1·06) | (0·99 to 1·01) | (1·31 to 1·49) | (98·58 to 98·62) |
| **Past** | 371 | 1743 | 1·17 | 0·991 | 1·64 | 98·61 |
|  |  |  | (1·05 to 1·31) | (0·985 to 0·998) | (1·47 to 1·83) | (98·6 to 98·62 |
| **Present** | 5240 | 29217 | 0·99 | 1·04 | 1·39 | 98·54 |
|  |  |  | (0·98 to 1) | (0·989 to 1·094) | (1·37 to 1·4) | (98·47 to 98·61) |
| * Patients without a diagnosis of depression before age 50 with (n=7517) and without (n=43183) a diagnosis of PD | | | | | | |
| ** Patients without a diagnosis of anxiety before age 50 with (n=7607) and without (n=43997) a diagnosis of PD | | | | | | |
| *** Male patients with (n=4859) and without (n=27684) a diagnosis of PD | | | | | | |
| ****Patients with specified smoking status with (n=7183) and without (n=40379) a diagnosis of PD | | | | | | |
| *****Patients with specified alcohol consumption history with (n=6653) and without (n=36711) a diagnosis of PD | | | | | | |
| Abbreviations: CI: confidence interval; LR: likelihood ratio; PPV: positive predictive value ;NPV: negative predicted value | | | | | | |
| For analysis for each age group and gender see supplementary material | | | | | | |
| LR+ = Sensitivity/(100-Specificity) :  ratio between the probability of a positive test result given the presence of the disease and the probability of a positive test result given the absence of the disease | | | | | | |
| LR- = (100-Sensitivity)/Specificity : ratio between the probability of a negative test result given the *presence* of the disease and the probability of a negative test result given the *absence* of the disease | | | | | | |

**Table 3. Corrected intercepts**

| **Males** |  |
| --- | --- |
| **50-59** | -6.64 |
| **60-69** | -5.85 |
| **70-79** | -5.32 |
| **80-89** | -5.06 |
| **90+** | -4.87 |
| **Females** |  |
| **50-59** | -7.65 |
| **60-69** | -6.54 |
| **70-79** | -5.7 |
| **80-89** | -5.51 |
| **90+** | -5.81 |

Calculation of **intercepts for each age-group/gender combination**

We first calculated the weighting required for each control so that the study sample matches the population prevalence. This is given by

$$w=\frac{cases}{controls}\times\frac{1-\pi}{\pi}$$

where *cases* and *controls* are the number of cases and controls respectively in the age-group/gender combination and $\pi$ is the prevalence of PD in the population for that combination. We then find the value *k* that needs to be subtracted from the estimated intercept in order to “fix” the predictions. That is, *k* is chosen so that

$$\frac{(cases\times\hat{\pi}_{cases}+w\times controls\times\hat{\pi}_{controls})}{(cases+w\times controls)}=\pi$$

where the average predicted risk for the cases ($\hat{\pi}_{cases}$) and controls ($\hat{\pi}_{controls}$) depend on *k*.

**Figure 1. Calibration slopes in the development and validation datasets.**

**Table 4. Multivariate logistic regression models for association of individual symptom presentations and risk factors (excluding tremor) with diagnosis of Parkinson's disease.**

| Covariate | Odds Ratio | 95% Conf. Interval | P-value |
| --- | --- | --- | --- |
| Balance problems | 2.89 | (2.36 to 3.54) | <0.001 |
| Cognitive decline | 1.93 | (1.11 to 3.37) | 0.020 |
| Constipation | 1.55 | (1.44 to 1.66) | <0.001 |
| Dizziness | 1.29 | (1.16 to 1.44) | <0.001 |
| Hypotension | 1.51 | (1.21 to 1.87) | <0.001 |
| Fatigue | 1.55 | (1.41 to 1.72) | <0.001 |
| Urinary dysfunction | 1.51 | (1.30 to 1.75) | <0.001 |
| Depression &/or anxiety | 1.63 | (1.50 to 1.77) | <0.001 |
| Memory problems | 1.48 | (1.18 to 1.84) | 0.001 |
| Rigidity | 12.13 | (8.34 to 17.65) | <0.001 |
| Hypersalivation | 7.66 | (3.60 to 16.33) | <0.001 |
| Smoking status |  |  |  |
| Non | 1 |  |  |
| Ex | 0.72 | (0.68 to 0.78) | <0.001 |
| Current | 0.43 | (0.39 to 0.48) | <0.001 |

**ROC curves without tremor**

Validation: AUC:0.66 (95%CI: 0.65-0.67)

Development: AUC:0.66 (95%CI: 0.65-0.67)

Calibration plots without tremor

**Table 5. Factors to be added in the calculation (without tremor) of predicted risk of PD to adjust for age group and gender.**

| **Males** |  |
| --- | --- |
| **50-59** | -4.54 |
| **60-69** | -3.48 |
| **70-79** | -2.78 |
| **80-89** | -2.49 |
| **90+** | -2.74 |
| **Females** |  |
| **50-59** | -5.45 |
| **60-69** | -4.26 |
| **70-79** | -3.1 |
| **80-89** | -3.05 |
| **90+** | -3.26 |

Table 6 LRs, PPV and NPV of individual prediagnostic symptom presentations and risk factors separately for the Development and the Validation samples

Development sample (n=16,476)

|  | **Number of patients with**  **Presentation** | |  |  |  |  |
| --- | --- | --- | --- | --- | --- | --- |
|  | PD | Controls | LR+ (95%CI) | LR- (95%CI) | PPV in percent (95%CI) | NPV in percent (95%CI) |
|  | 2399 | 14077 |  |  |  |  |
| Tremor | 969 | 93 | 61.14 (49.64 to 75.30) | 0.600 (0.581 to 0.620) | 46.47 (41.34 to 51.67) | 99.16 (99.13 to 99.18) |
| Constipation | 925 | 3176 | 1.71 (1.61 to 1.81) | 0.793 (0.768 to 0.820) | 2.37 (2.24 to 2.51) | 98.89 (98.85 to 98.92) |
| Depression* | 398 | 1221 | 1.90 (1.72 to 2.11) | 0.906 (0.888 to 0.925) | 2.63 (2.38 to 2.91) | 98.73 (98.70 to 98.76) |
| Fatigue | 316 | 1146 | 1.62 (1.44 to 1.82) | 0.945 (0.930 to 0.961) | 2.25 (2.00 to 2.52) | 98.68 (98.65 to 98.70) |
| Dizziness | 353 | 1321 | 1.57 (1.41 to 1.75) | 0.941 (0.925 to 0.958) | 2.18 (1.96 to 2.42) | 98.68 (98.66 to 98.70) |
| Anxiety** | 260 | 906 | 1.69 (1.48 to 1.92) | 0.949 (0.934 to 0.964) | 2.34 (2.06 to 2.66) | 98.67 (98.65 to 98.69) |
| Shoulder pain/stiffness | 294 | 1217 | 1.42 (1.26 to 1.60) | 0.960 (0.945 to 0.976) | 1.97 (1.75 to 2.22) | 98.65 (98.63 to 98.68) |
| Erectile dysfunction *** | 122 | 670 | 1.08 (0.90 to 1.30) | 0.993 (0.976 to 1.010) | 1.51 (1.26 to 1.81) | 98.61 (98.59 to 98.63) |
| Insomnia | 175 | 793 | 1.29 (1.11 to 1.52) | 0.982 (0.971 to 0.994) | 1.81 (1.55 to 2.11) | 98.62 (98.61 to 98.64) |
| Neck pain/stiffness | 162 | 916 | 1.04 (0.88 to 1.22) | 0.997 (0.986 to 1.009) | 1.45 (1.24 to 1.70) | 98.60 (98.59 to 98.62) |
| Urinary dysfunction | 153 | 429 | 2.09 (1.75 to 2.50) | 0.966 (0.955 to 0.976) | 2.89 (2.42 to 3.43) | 98.65 (98.63 to 98.66) |
| Balance problems | 104 | 202 | 3.02 (2.39 to 3.81) | 0.971 (0.962 to 0.979) | 4.11 (3.29 to 5.13) | 98.64 (98.63 to 98.65) |
| Memory problems | 77 | 214 | 2.11 (1.63 to 2.73) | 0.983 (0.975 to 0.990) | 2.91 (2.27 to 3.73) | 98.62 (98.61 to 98.63) |
| Hypotension | 77 | 189 | 2.39 (1.84 to 3.10) | 0.981 (0.974 to 0.988) | 3.28 (2.55 to 4.22) | 98.63 (98.62 to 98.64) |
| Rigidity | 56 | 28 | 11.74 (7.47 to 18.43) | 0.979 (0.973 to 0.985) | 14.28 (9.59 to 20.74) | 98.63 (98.62 to 98.64) |
| Cognitive decline | 12 | 27 | 2.61 (1.32 to 5.14) | 0.997 (0.994 to 1.000) | 3.57 (1.84 to 6.80) | 98.60 (98.60 to 98.61) |
| Hypersalivation | 8 | 6 | 7.82 (2.72 to 22.53) | 0.997 (0.995 to 0.999) | 10.00 (3.71 to 24.24) | 98.60 (98.60 to 98.61) |
| Anosmia | 5 | 17 | 1.73 (0.64 to 4.67) | 0.999 (0.997 to 1.001) | 2.39 (0.90 to 6.22) | 98.60 (98.60 to 98.60) |
| Apathy | 1 | 3 | 1.96 (0.20 to 18.80) | 1.000 (0.999 to 1.001) | 2.70 (0.29 to 21.07) | 98.60 (98.60 to 98.60) |
| Smoking status**** |  |  |  |  |  |  |
| Never | 1275 | 6277 | 1.16 (1.12 to 1.21) | 0.828 (0.784 to 0.875) | 1.62 (1.56 to 1.69) | 98.84 (98.77 to 98.90) |
| Past | 654 | 3650 | 1.02 (0.96 to 1.10) | 0.989 (0.960 to 1.020) | 1.43 (1.34 to 1.54) | 98.61 (98.57 to 98.66) |
| Present | 200 | 2247 | 0.51 (0.44 to 0.58) | 1.111 (1.093 to 1.129) | 0.72 (0.63 to 0.82) | 98.45 (98.42 to 98.47) |
| Alcohol consumption***** |  |  |  |  |  |  |
| Never | 108 | 523 | 1.16 (0.95 to 1.42) | 0.992 (0.981 to 1.004) | 1.62 (1.33 to 1.97) | 98.61 (98.60 to 98.63) |
| Past | 321 | 1696 | 1.06 (0.95 to 1.18) | 0.989 (0.968 to 1.010) | 1.48 (1.33 to 1.65) | 98.62 (98.59 to 98.64) |
| Present | 1539 | 8815 | 0.98 (0.95 to 1.00) | 1.084 (0.989 to 1.188) | 1.37 (1.34 to 1.41) | 98.48 (98.34 to 98.62) |
| * Patients without a diagnosis of depression before age 50 with (n=2220) and without (n=12971) a diagnosis of PD | | | | | | |
| ** Patients without a diagnosis of anxiety before age 50 with (n=2237) and without (n=13159) a diagnosis of PD | | | | | | |
| *** Male patients with (n=1414) and without (n=8390) a diagnosis of PD | | | | | | |
| ****Patients with specified smoking status with (n=2129) and without (n=12174) a diagnosis of PD | | | | | | |
| *****Patients with specified alcohol consumption history with (n=1968) and without (n=11034) a diagnosis of PD | | | | | | |

Validation sample (n=38,645)

|  | **Number of patients with**  **Presentation** | |  |  |  |  |
| --- | --- | --- | --- | --- | --- | --- |
|  | PD | Controls | LR+ (95%CI) | LR- (95%CI) | PPV in percent (95%CI) | NPV in percent (95%CI) |
| Total | 5967 | 32678 |  |  |  |  |
| Tremor | 2409 | 251 | 54.38 (47.90 to 61.74) | 0.587 (0.574 to 0.600) | 43.57 (40.48 to 46.72) | 99.17 (99.16 to 99.19) |
| Constipation | 2057 | 7488 | 1.56 (1.50 to 1.62) | 0.835 (0.818 to 0.851) | 2.16 (2.08 to 2.25) | 98.83 (98.81 to 98.85) |
| Depression* | 980 | 2984 | 1.87 (1.75 to 2.00) | 0.904 (0.892 to 0.916) | 2.59 (2.43 to 2.76) | 98.73 (98.72 to 98.75) |
| Fatigue | 891 | 2645 | 1.91 (1.78 to 2.05) | 0.920 (0.909 to 0.931) | 2.64 (2.46 to 2.83) | 98.71 (98.70 to 98.73) |
| Dizziness | 826 | 3006 | 1.56 (1.45 to 1.67) | 0.944 (0.933 to 0.954) | 2.16 (2.02 to 2.32) | 98.68 (98.66 to 98.69) |
| Anxiety** | 658 | 2170 | 1.73 (1.59 to 1.88) | 0.944 (0.934 to 0.954) | 2.40 (2.21 to 2.60) | 98.68 (98.66 to 98.69) |
| Shoulder pain/stiffness | 684 | 3011 | 1.29 (1.19 to 1.39) | 0.971 (0.961 to 0.981) | 1.79 (1.66 to 1.94) | 98.64 (98.63 to 98.65) |
| Erectile dysfunction *** | 332 | 1536 | 1.21 (1.08 to 1.36) | 0.982 (0.970 to 0.993) | 1.69 (1.51 to 1.89) | 98.63 (98.61 to 98.64) |
| Insomnia | 432 | 1889 | 1.30 (1.17 to 1.43) | 0.982 (0.974 to 0.990) | 1.81 (1.64 to 1.99) | 98.63 (98.61 to 98.64) |
| Neck pain/stiffness | 388 | 2218 | 0.99 (0.89 to 1.10) | 1.001 (0.993 to 1.008) | 1.39 (1.25 to 1.54) | 98.60 (98.59 to 98.61) |
| Urinary dysfunction | 345 | 987 | 1.98 (1.76 to 2.23) | 0.969 (0.963 to 0.976) | 2.74 (2.44 to 3.07) | 98.64 (98.63 to 98.65) |
| Balance problems | 296 | 417 | 4.02 (3.47 to 4.66) | 0.961 (0.955 to 0.967) | 5.40 (4.70 to 6.20) | 98.65 (98.65 to 98.66) |
| Memory problems | 180 | 536 | 1.90 (1.61 to 2.25) | 0.985 (0.980 to 0.990) | 2.63 (2.24 to 3.09) | 98.62 (98.61 to 98.63) |
| Hypotension | 178 | 433 | 2.33 (1.96 to 2.77) | 0.982 (0.977 to 0.987) | 3.20 (2.71 to 3.78) | 98.62 (98.62 to 98.63) |
| Rigidity | 172 | 68 | 14.33 (10.84 to 18.95) | 0.972 (0.968 to 0.977) | 16.91 (13.34 to 21.20) | 98.64 (98.63 to 98.64) |
| Cognitive decline | 24 | 52 | 2.62 (1.61 to 4.24) | 0.997 (0.996 to 0.999) | 3.58 (2.24 to 5.68) | 98.60 (98.60 to 98.61) |
| Hypersalivation | 28 | 17 | 9.33 (5.11 to 17.04) | 0.996 (0.994 to 0.997) | 11.70 (6.77 to 19.48) | 98.61 (98.60 to 98.61) |
| Anosmia | 25 | 49 | 2.89 (1.79 to 4.68) | 0.997 (0.995 to 0.999) | 3.94 (2.47 to 6.23) | 98.60 (98.60 to 98.61) |
| Apathy | 3 | 10 | 1.70 (0.47 to 6.17) | 1.000 (0.999 to 1.000) | 2.36 (0.66 to 8.06) | 98.60 (98.60 to 98.60) |
| Smoking status**** |  |  |  |  |  |  |
| Never | 3121 | 14312 | 1.22 (1.19 to 1.25) | 0.776 (0.748 to 0.806) | 1.70 (1.66 to 1.74) | 98.91 (98.87 to 98.95) |
| Past | 1422 | 8546 | 0.93 (0.89 to 0.97) | 1.031 (1.012 to 1.051) | 1.30 (1.24 to 1.36) | 98.56 (98.53 to 98.58) |
| Present | 511 | 5347 | 0.53 (0.49 to 0.58) | 1.109 (1.097 to 1.121) | 0.75 (0.69 to 0.82) | 98.45 (98.43 to 98.47) |
| Alcohol consumption***** |  |  |  |  |  |  |
| Never | 263 | 1220 | 1.18 (1.04 to 1.34) | 0.991 (0.984 to 0.998) | 1.65 (1.45 to 1.87) | 98.61 (98.60 to 98.62) |
| Past | 721 | 4055 | 0.97 (0.91 to 1.05) | 1.005 (0.991 to 1.018) | 1.36 (1.27 to 1.47) | 98.59 (98.57 to 98.61) |
| Present | 3701 | 20402 | 0.99 (0.98 to 1.01) | 1.022 (0.962 to 1.086) | 1.39 (1.37 to 1.41) | 98.57 (98.48 to 98.65) |
| * Patients without a diagnosis of depression before age 50 with (n=5297) and without (n=30212) a diagnosis of PD | | | | | | |
| ** Patients without a diagnosis of anxiety before age 50 with (n=5370) and without (n=30651) a diagnosis of PD | | | | | | |
| *** Male patients with (n=3445) and without (n=19294) a diagnosis of PD | | | | | | |
| ****Patients with specified smoking status with (n=5054) and without (n=28205) a diagnosis of PD | | | | | | |
| *****Patients with specified alcohol consumption history with (n=4685) and without (n=25677) a diagnosis of PD | | | | | | |
